# Share to Seek: The Effects of Disease Complexity on Health Information–Seeking Behavior

**DOI:** 10.2196/21642

**Published:** 2021-03-24

**Authors:** Ashwag Alasmari, Lina Zhou

**Affiliations:** 1 Computer Science Department King Khalid University Abha Saudi Arabia; 2 University of Maryland, Baltimore County Baltimore, MD United States; 3 University of North Carolina at Charlotte Charlotte, NC United States

**Keywords:** health information consumers, multimorbidity, information searching, information seeking, disease development

## Abstract

**Background:**

Web-based question and answer (Q&A) sites have emerged as an alternative source for serving individuals’ health information needs. Although a number of studies have analyzed user-generated content in web-based Q&A sites, there is insufficient understanding of the effect of disease complexity on information-seeking needs and the types of information shared, and little research has been devoted to the questions concerning multimorbidity.

**Objective:**

This study aims to investigate seeking of health information in Q&A sites at different levels of disease complexity. Specifically, this study investigates the effects of disease complexity on information-seeking needs, types of information shared, and stages of disease development.

**Methods:**

First, we selected a random sample of 400 questions separately from each of the Q&A sites: Yahoo Answers and WebMD Answers. The data cleaning resulted in a final set of 624 questions from the two sites. We used a mixed methods approach, including qualitative content analysis and quantitative statistical analysis.

**Results:**

The one-way results of ANOVA showed significant effects of disease complexity (single vs multimorbid disease questions) on two information-seeking needs: diagnosis (*F*_1,622_=5.08; *P*=.02) and treatment (*F*_1,622_=4.82; *P*=.02). There were also significant differences between the two levels of disease complexity in two stages of disease development: the general health stage (*F*_1,622_=48.02; *P*<.001) and the chronic stage (*F*_1,622_=54.01; *P*<.001). In addition, our results showed significant effects of disease complexity across all types of shared information: demographic information (*F*_1,622_=32.24; *P*<.001), medical diagnosis (*F*_1,622_=11.04; *P*<.001), and treatment and prevention (*F*_1,622_=14.55; *P*<.001).

**Conclusions:**

Our findings present implications for the design of web-based Q&A sites to better support health information seeking. Future studies should be conducted to validate the generality of these findings and apply them to improve the effectiveness of health information in Q&A sites.

## Introduction

### Background

Reports from the Pew Research Center indicate that an increasing number of people use web-based services to obtain health information, rising from 25% of Americans in 2000 to 72% in 2014 [1]. Users look for specific diseases and treatments or other people with similar health problems, usually to diagnose themselves or others. These web-based resources range from general search engines to specific sites devoted to health information. One potential resource that could meet the health information needs of many internet users is question and answer (Q&A) sites.

Web-based Q&A sites allow health information consumers (HICs) to post questions for other users to answer [2]. Q&A sites may focus on specific topics or be more general in nature. Another characteristic distinction between different types of Q&A sites is whether the posted answers are curated by experts (expert curated) or by other users (community based). In an expert-curated site, experts are considered an essential part of the community and their answers are ranked first, followed by answers posted by other contributors. In a community-based site, HICs seek support from peers with similar conditions, and the answers are featured typically based on the total number of community votes.

The motives of users participating in Q&A sites, especially in the long term, have been explained based on social theories [3]. According to the social exchange theory, individuals exchange valuable resources through contacts to receive something that benefits both parties [4]. This reciprocity encourages social interactions in a co-operative fashion between individuals, even when the rewards are not tangible. Individuals may also share information with someone believing that a third party could offer similar help in the future when needed, and this is termed generalized reciprocity [3]. Beyond finding solutions to their problems, those who seek information possess other benefits such as meta-knowledge (becoming aware of further resources such as people, databases, or documents), problem reformulation, acknowledgment and legitimation from respected people, and validation of their plans or solutions [5]. HICs who participate in web-based Q&A communities want to better understand their condition and receive treatment options from professionals [6], evaluate their health with regard to risks and prevention of further disease [6], or communicate with patients who share similar experiences [7].

Several studies have examined the information-seeking behaviors of HICs based on the characteristics of their questions and information needs [8,9]. A study examining cancer-related topics on Yahoo Answers found that HICs provided rich information about their problems, emotions, social relationships, and life situations in their questions [8]. The findings not only highlighted the complexity of health-related issues but also indicated that there are multiple reasons why people would search on the web for answers. In HICs with cancer, for example, those who post during their cancer diagnosis or treatment ask mainly for advice. Survivors seemed to share personal narratives, and terminal patients sought acknowledgment and validation of their choices [8]. In addition, previous studies have demonstrated that the language used by HICs is based not only on controlled vocabularies developed for lay consumers, such as consumer health vocabularies, but also on the terminology used by health professionals, such as SNOMED Clinical Terms [9,10]. Another stream of research focused on understanding web-based health questions by extracting the linguistic features of the text. For example, a study examined health questions on the topic of eating disorders in Yahoo! Answers for linguistic style and sentiment [11].

One major limitation of previous studies is that they focused on single-disease entities in isolation, such as cancer [8,12,13] or diabetes [9]. These are complex diseases that require careful management, especially when they are associated with the presence of other diseases. The population of patients with multiple chronic conditions, also known as multimorbidity, has increased immensely over the past few decades [14]. Multimorbidity negatively impacts a patient’s quality of life, hospitalization, and mortality [15]. Patients with multimorbidity face unique challenges because they are expected to distinguish between the symptoms of different diseases and make sense of the abundance of information obtained from different web-based health resources [16,17]. Despite the rapid growth of online health communities, most of these communities cater to the management of single chronic diseases and are not responsive to the needs of HICs with multimorbidity [16,18,19]. The effect of disease complexity in its entire spectrum (single and multiple diseases) on information-seeking needs and types of information shared by HICs appears to be only partially investigated in the literature.

### Objectives

This study aims to investigate health information sharing in web-based Q&A platforms with a specific focus on the effect of disease complexity, namely the characteristics of multiple-disease health questions as compared with characteristics of single-disease health questions. There is little understanding of the effect of disease complexity on the characteristics of health questions, and we do not know how deeply these differences run. More specifically, this paper aims to answer the following research questions: Are there any differences between questions relating to a single disease compared with those relating to multimorbidity in web-based Q&A platforms? How deep do these differences run in terms of information-seeking needs, types of information shared, and stages of disease development?

## Methods

### Disease Topic Selection

We chose kidney disease as the health topic. According to statistics from the National Institute of Diabetes and Digestive and Kidney Diseases, kidney disease ranges from simple conditions, such as small stones, to more serious illnesses such as chronic kidney disease, the prevalence of which is estimated to be 14% in the general population [20]. Chronic kidney disease has significant complications such as high blood pressure, cardiovascular disease, anemia, weak bones, poor nutritional health, and neuropathy [21]. At the same time, other chronic conditions increase the risk of chronic kidney disease, such as diabetes and high blood pressure. Thus, multimorbidity among patients with kidney disease is extremely high, which would inevitably have a significant impact on their ability to identify, evaluate, and manage their health [22-26].

### Q&A Sites

We collected data from an expert-curated Q&A site, WebMD Answers, and a community-based site, Yahoo! Answers. WebMD is one of the most influential web-based health sites [27,28], and users were able to post questions for certified health experts to answer in the Q&A section, which covered more than 900 health topics. We collected all posts from August 2008 until the closure of WebMD Answers in 2018 and its successor, that is, the Questions & Answers A-Z section that offers preset answers and questions.

Yahoo! Answers features health as one of the top-level categories; therefore, it was selected as a community-based Q&A site. Since its creation in December 2005, Yahoo! Answers has become a popular internet reference site worldwide, and it is the most frequented community Q&A site in the United States. As of June 2019, the site ranked ninth in global internet traffic and engagement over the past 90 days and seventh in the United States [29]. We collected data from this site for a period of 9 years (2006-2015).

### Sampling Process, Data Collection, and Preparation

Given the selection of kidney disease, we screened questions based on the following key terms: kidney, kidney infection, kidney stone, kidney cancer, kidney disease, chronic kidney disease, dialysis, kidney failure, renal artery stenosis, and renal cell carcinoma. These key terms were selected as they directly refer to kidney conditions or early signs of chronic kidney disease [30]. Using an application programming interface, we sampled 400 random questions from Yahoo! Answers and 400 questions from WebMD related to the abovementioned kidney-related key terms.

We manually removed noise such as advertisements, irrelevant questions (non–kidney-related, nonhuman subjects, or student projects), or posts that did not have an actual question. After cleaning, we had 316 questions from Yahoo Answers and 308 from WebMD Answers. In total, the data cleaning process resulted in a final set of 624 questions. For each of the included questions, we extracted the title, descriptions, date of posting, categories under which the questions were posted, number of answers, and the answers themselves.

The data sets contained both qualitative (the actual content) and quantitative information (such as dates of posting and replies, demographics of participants, topic, and length and number of questions). In this study, we focused on the questions themselves. Therefore, a mixed methods approach was necessary for this analysis.

Content analysis is widely used as a qualitative research method for analyzing questions and answers from web-based Q&A websites [3]. The content of each post is coded into a set of categories, and analysis is performed based on these categories and their frequencies. In this study, we used directed content analysis, where the analysis started with findings from previous research as guidance for the initial codes [31]. The qualitative data were transformed into quantitative data through deductive analysis based on predetermined frameworks and themes and were then analyzed quantitatively. Quantitative data analysis allowed for comparisons of groups and for examining the effect of covariates, in this case, disease complexity.

### Dependent Variables and Independent Variables

The independent variable was disease complexity, with two levels: single and multiple (multimorbidity). We measured the variables based on the number of diseases described in the health questions. The health question was labeled as *single* if it was related to a single disease (kidney only) and otherwise labeled as *multiple*. We considered the following dependent variables: information-seeking needs, stages of disease development, and types of shared information.

We contextualized the health questions by adapting relevant findings from a previous study on health information seeking [32]. The three main areas of interest for our analysis included health information–seeking needs, stages of disease development, and information shared (personal and medical). Importantly, in recognition that chronic diseases are more complex, we introduced a comprehensive disease development stage by adapting a framework of chronic illness development [33]. We coded the stages of disease development using the following Zhang and Corbin frameworks, and the coders were asked to decide which disease stage the health questions are related to. The information-seeking needs and types of information shared included several variables coded as binary (present or absent). The following are detailed descriptions of our coding schema.

#### Information-Seeking Needs

HICs ask health-related questions to address specific information needs [32]:

- Symptom: to gain an understanding of the symptoms of a kidney or any other related disease.
- Diagnosis: to confirm the nature of a certain disease.
- Causes: to figure out the causes of the disease.
- Prognoses: to inquire about the hypothetical effect of a disease.
- Treatment: to explore treatment alternatives to kidney disease.
- Supplements and lifestyle: to explore lifestyle and diet in people with kidney disease and use different supplements.
- Information sources, medical profession, and related types of information: to look for medical experts in the field and any kind of resources to fulfill HIC information needs.
- Drug interaction: to ask for more details about unfavorable and unexpected signs, symptoms, or diseases associated with the use of a drug without any judgment about the causality or relationship to drug use.
- Similar experiences: to connect to patients with similar conditions.

#### Stage of Disease Development

Diagnosing and treating a disease or condition is an ongoing process. HICs at different stages in this process often have different levels of information needs [13]. HICs may also display different information-seeking behaviors based on the nature and extent of their needs. We adopted a disease development model consisting of 8 stages [32]: (1) being healthy; (2) self-diagnosed as being ill; (3) before having a medical test or checkup; (4) after being diagnosed or self-diagnosed as ill; (5) before treatment (such as surgery or medication); (6) during treatment (including medications or exercise); (7) after treatment; and (8) when the disease becomes chronic or reaches the terminal stage.

Multimorbidity is strongly associated with chronic disease. Accordingly, we also drew on the stages of chronic illness in understanding the questions of HICs who are chronically ill. To this end, stages of disease development were extended to include the chronic illness trajectory framework [33] for clinician use in nursing care and chronic illness management. This trajectory framework was built based on the idea that the course of chronic conditions varies and changes over time. It consists of 9 stages [33]: (1) pretrajectory, before disease onset; (2) trajectory onset, appearance of symptoms and diagnosis; (3) stability, condition and symptoms are under control. Everyday life is unaffected, illness management is home-centered, and hospitalization is not required; (4) unstable, condition and symptoms are not under control. Everyday life is disrupted. However, care remains to be centered at home; (5) acute, symptoms or complications require hospitalization or other measures. Everyday life activities are cut back or severely curtailed; (6) crisis, a life-threatening situation that requires emergency care. Everyday life is placed on hold; (7) comeback, a return to everyday life activities, possibly with changed ability for everyday life activities; (8) downward, decline associated with increased disability and trouble controlling symptoms, requires adaptation in everyday life activities; and (9) dying, death of the patient.

#### Types of Information Shared

HICs provide demographic and medical information in their questions that represent their understanding of their diseases and communicate their information needs to others [32]. Demographic information included age, gender, ethnicity, weight, location, and profession. Medical information includes symptoms, medical tests, treatment, time of treatment, lifestyle, drugs, personal and family medical history, insurance, and time in hospital.

To facilitate question analysis, we developed a web-based annotation system. Two annotators coded the questions independently, with one having a medical degree. Each coder was asked to determine whether any of the categories in the coding schema was present or absent in the question. The questions were split randomly between the coders with 20% overlap to check for intercoder agreement. A comparison between the two sets of coding results showed that the intercoder agreement over the overlapping data was 87.9%. Discrepancies in the coding results were discussed and resolved among the coders, who then used the results of this discussion to review and revise the overall questions until they reached an agreement.

## Results

### Descriptive Statistics

Descriptive statistics for the data sets are presented in Table 1. Among the questions, 85.1% (531/624) involved single diseases and 14.9% (93/624) involved multimorbidity. Specifically, the single-disease questions accounted for approximately 50.6% (316/624) of questions from Yahoo! Answers and 49.3% (308/624) questions from WebMD Answers, respectively; multimorbid accounted for approximately 8.0% (50/624) of questions on Yahoo! Answers and 6.9% (43/624) on WebMD Answers, respectively. We performed one-way analysis of variance (ANOVA) to test the effect of disease complexity on the selected characteristics of the posted questions. The analysis was followed up with Tukey honestly significant difference test to examine the differences between different values of the variables.

**Table 1:** Descriptive statistics of the data sets and analysis of variance results of disease complexity.

Variable		Single (n=531; 85.1%), mean (SD)	Multimorbidity (n=93; 14.9%), mean (SD)	*F* test (*df*)	*P* value
**Types of information needs**					
	Symptom	0.12 (0.33)	0.08 (0.27)	1.72 (1,622)	.19
	Cause	0.19 (0.39)	0.14 (0.35)	1.16 (1,622)	.28
	Diagnose	0.22 (0.41)	0.12 (0.32)	5.08 (1,622)	.02^a^
	Treatment	0.16 (0.37)	0.26 (0.44)	4.82 (1,622)	.02^a^
	Prognoses	0.13 (0.33)	0.17 (0.38)	1.31 (1,622)	.25
	Drug	0.04 (0.19)	0.06 (0.25)	1.42 (1,622)	.23
	Lifestyle	0.13 (0.34)	0.15 (0.36)	0.29 (1,622)	.59
	Similar	0.03 (0.17)	0.05 (0.23)	1.66 (1,622)	.19
	Source	0.06 (0.23)	0.09 (0.28)	1.03 (1,622)	.31
	Others	0.23 (0.42)	0.28 (0.45)	1.26 (1,622)	.26
**Stages of disease development**					
	Health stages of questions	3.92 (3.43)	6.51 (2.59)	48.02 (1,622)	<.001^b^
	Chronic stages	1.04 (1.6)	2.4 (1.87)	54.01 (1,622)	<.001^b^
**Type of personal information shared**					
	Demographic	0.21 (0.41)	0.48 (0.5)	32.24 (1,622)	<.001^b^
	Medical diagnosis	0.4 (0.49)	0.58 (0.5)	11.04 (1,622)	<.001^b^
	Treatment and prevention	0.25 (0.43)	0.44 (0.5)	14.55 (1,622)	<.001^b^

^a^Significant as *P*<.05.

^b^Significant as *P*<.001.

### Types of Information Needs

The ANOVA results (Table 1) showed a significant effect of disease complexity on two information-seeking needs: diagnosis (*F*_1,622_=5.08; *P*=.02) and treatment (*F*_1,622_=4.82; *P*=.02). However, disease complexity did not have any effect on other types of information-seeking needs such as symptoms (*P*=.19), causes (*P*=.28), prognosis (*P*=.25), drug interactions (*P*=.23), lifestyle (*P*=.59), similar experiences (*P*=.19), source (*P*=.31), and others (*P*=.26).

The post-hoc comparison showed that the single-disease questions tend to include more diagnostic information (mean 0.22, SD 0.41; *P*=.02 than the multimorbid counterpart, whereas the multimorbid questions tend to contain more treatment-related information than single-disease ones (mean 0.26, SD 0.44; *P*=.02).

### Stages of Disease Development

There were statistically significant differences in the health stage (*F*_1,622_=48.02; *P*<.001) and chronic stage (*F*_1,622_=54.01; *P*<.001) between the two levels of disease complexity. Specifically, multimorbid questions included questions more frequently at health stages of questions (mean 6.51, SD 2.59; *P*<.001) and at advanced or chronic stages of illness (mean 2.4, SD 1.87; *P*<.001).

### Types of Information Shared

The ANOVA results revealed the effects of disease complexity on the types of information shared, including demographic information (*F*_1,622_=32.24; *P*<.001), medical diagnosis (*F*_1,622_=11.04; *P*<.001*),* and treatment and prevention (*F*_1,622_=14.55; *P*<.001). Specifically, questions from multimorbid HICs are more likely to provide demographic information such as age, ethnicity, and weight (mean 0.48, SD 0.5; *P*<.001) in their questions compared with single-disease HICs (mean 0.21, SD 0.41; *P*<.001). In addition, multimorbid HICs are more likely to include information relating to diagnosis (mean 0.58, SD 0.5; *P*<.001) and information relating to treatment and prevention (mean 0.44, SD 0.5; *P*<.001).

**Figure 1:**
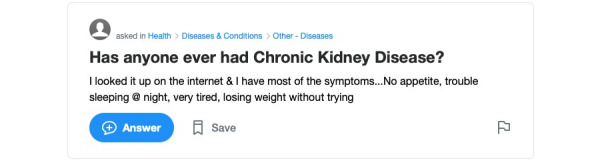
Illustrations of information seeking and sharing behavior in single-disease health questions.

**Figure 2:**
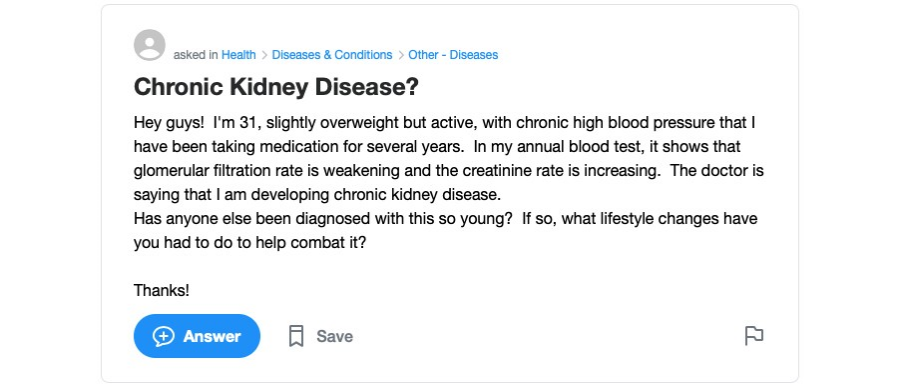
Illustrations of information seeking and sharing behavior in multi-disease health questions.

Figures 1 and 2 present two illustrations of information sharing in health questions. In the single-disease question (Figure 1), the HIC sought a possible diagnosis by sharing a list of symptoms. However, the question contained a minimal amount of demographic and medical information including medical diagnosis or information relating to treatment and prevention. In the multidisease question (Figure 2), the HIC shared some personal information, including demographics, medical diagnosis, treatment, and multimorbidity, and is seeking very specific information relating to diagnosis and lifestyle.

## Discussion

### Principal Findings and Explanations

This research aims to investigate whether disease complexity affects information-seeking needs, stages of disease development, and the type of shared information on Q&A sites. Our empirical data analysis results revealed several significant effects.

Questions relating to single diseases were more likely to include questions about diagnosis when compared with multimorbid questions. This is not in line with medical care assumptions that underdiagnoses are a real threat to managing patients with multiple chronic conditions [34], who may not be allocated sufficient time for assessment in clinics. The results are also surprising if we consider the challenges involved in making sense of the plethora of symptoms and the complex interaction between different diseases. The reasons behind this are not clear, as there are no differences in the number of question posts asking about two related topics: symptoms and causes. Disease complexity was not found to have an effect on any other type of information needs, including symptoms, causes, prognoses, drugs, lifestyle, similar experiences, sources, and others. Potentially, patients with multimorbidity may have a better understanding of the diagnostic pathways and are content with the information they already know. In addition, the finding of this study supports that multimorbid HICs asking about treatment more frequently than single-disease HICs. This is in line with previous research assuming that people with major illnesses, such as cancer, ask mainly for advice; survivors seem to share personal narratives; and terminal patients seek acknowledgment and validation of their choices [8]. Similarly, multiple chronic conditions may require more complex management.

Another major finding is the disease stage. Multimorbid questions focused more frequently on advanced stages of disease development. We provide several alternative explanations for this finding. First, HICs at a later stage of a chronic disease may need more information to validate their choices, such as looking for detailed clinical information, which experts cannot do for various reasons. Patients might understand that care is patient centered, and it is common for treatments to differ, especially across different states or countries. In addition, patients at later stages may require more general information, such as lifestyle, for which peers can serve as great information sources.

Disease complexity was found to play a major role in determining the types of information shared on web-based Q&A sites. In particular, the multimorbid HICs included more demographic and medical information in their questions, which included information related to diagnosis and information relating to treatment and prevention. Although the management of chronic disease is a highly collaborative process between patients and providers, the work that patients must take on during the various stages of chronic disease progression is immense [35,36]. Multimorbid HICs ask for additional information about management and treatments of their diseases, further highlighting their active involvement in health self-management.

### Contribution and Design Implications

This study makes several novel scientific contributions to health consumer informatics. To the best of our knowledge, this is the first study to empirically investigate the effects of disease complexity on the types of information shared in two Q&A sites. In addition, this is the first study to examine the disease stages of HICs with respect to disease complexity. Overall, this study provides a comprehensive examination of a wide variety of question characteristics, which is unique among studies on web-based health Q&A sites.

This study used mixed methods that combine qualitative and quantitative methods to understand health information–seeking behavior in web-based Q&A sites. In addition, the study considered two common types of Q&A sites, community based and expert curated, for analyzing health information–seeking behavior.

Unlike previous studies that view information sharing and information seeking as two conflicting goals [37-39], the findings of this study suggest that information sharing can facilitate information seeking in online health communities. Previous studies have focused more on individual motivations and intentions to share information but less on the actual sharing behaviors and the content of sharing [40]. In addition, we extended previous theories of information-seeking behavior by accounting for the dynamics of information needs; in other words, the process of information seeking can vary with the stages of disease development.

Our research findings have several implications for improving web-based Q&A sites. The information needs of HICs with chronic diseases may also change over time as their disease evolves, such as substantial disruptions to their everyday lives. As a result, effective support from peers who share similar characteristics and experiences would be very helpful. It is worth noting that HICs with a chronic disease grow their knowledge as they continuously manage their conditions and take more responsibility for their illnesses [33]. However, there is a lack of research investigating how the use of web-based information evolves as HICs gain experience moving through a chronic disease trajectory. How to design systems for HICs with chronic diseases that can not only support them in managing their own evolving health conditions and related knowledge but also enable them to help others with similar conditions are important questions for future research.

This study provides strong evidence that multimorbid HICs share both demographic and medical information when they seek health information on the web. Although web-based Q&A sites encourage the exchange of a significant amount of health information, these sites can benefit from improving the organization of information for community reuse. For instance, these sites could better support HICs with specific information needs by organizing the questions based on specific types of information needs, such as diagnosis, treatment, and side effects, and by encouraging them to share their demographic and medical information without compromising their personal privacy.

### Limitations and Future Directions

Our study has several limitations. As far as the data sources are concerned, we collected health questions from two types of Q&A sites. Thus, caution should be exercised when generalizing the findings to other Q&A sites. For instance, Twitter has been used for Q & A in the health context. It would help enrich the Q&A literature to build a tweets data set on health Q&A and use it to validate the findings of this study. In addition, multiple disease questions had a much smaller proportion as opposed to a single-disease question in this study. Furthermore, we have chosen to focus on health topics on the kidney disease. Although the findings of this study are expected to be extensible to other chronic diseases, they still require empirical validations. In addition to the health questions, which is the focal point of the analysis in this study, the Q&A sites also provide other types of information such as user profiles, comments, answers, and ratings. Integrating information from these multiple dimensions is expected to achieve a deeper understanding of the web-based behavior of HICs. On the other hand, the disclosures of increasing amount of personal health information may raise privacy concerns. Thus, how HIC trade-off between information needs and information disclosure is an interesting question for future research. The qualitative approach used in this study helps uncover the contextual characteristics of questions, which however is difficult to scale. Text mining has the potential to address the limitation by automating this process. The data generated through this study can serve as the training data for building text-mining models.

### Conclusions

Multiple disease or multimorbidity questions seem to play a major role in the stages of disease development and types of information shared, highlighting a deeper understanding of the complexities of their conditions. Regarding the types of information needs, multimorbidity has a minor implication related to treatment. It is also the case of single-disease questions that seem to be relevant only for types of information needs in terms of diagnosis. This study is a valuable first step in investigating the effects of multimorbidity on different types of information shared in two Q&A sites. The findings present implications for designing web-based Q&A sites to better support health information seeking. 

## Abbreviations

ANOVA: analysis of variance
HIC: health information consumer
Q&A: question and answer
