# Correction: Share to Seek: The Effects of Disease Complexity on Health Information–Seeking Behavior

**DOI:** 10.2196/42949

**Published:** 2022-09-30

**Authors:** Ashwag Alasmari, Lina Zhou

**Affiliations:** 1 Computer Science Department King Khalid University Abha Saudi Arabia; 2 University of Maryland, Baltimore County Baltimore, MD United States; 3 University of North Carolina at Charlotte Charlotte, NC United States

In “Share to Seek: The Effects of Disease Complexity on Health Information–Seeking Behavior” (J Med Internet Res 2021;23(3):e21642), the authors noted two errors.

In the originally published article, affiliations of the first author, Ashwag Alasmari, appeared in the following order:

Ashwag Alasmari^1,2^

^1^University of Maryland, Baltimore County, Baltimore, MD, United States

^2^King Khalid University, Abha, Saudi Arabia

Moreover, the original Affiliation 2 inadvertently did not specify any department.

In the corrected version, affiliations appear in the following order, and the reordered Affiliation 1 specifies the author's affiliated department:

Ashwag Alasmari^1,2^

^1^Computer Science Department, King Khalid University, Abha, Saudi Arabia

^2^University of Maryland, Baltimore County, Baltimore, MD, United States

The correction will appear in the online version of the paper on the JMIR Publications website on September 30, 2022, together with the publication of this correction notice. Because this was made after submission to PubMed, PubMed Central, and other full-text repositories, the corrected article has also been resubmitted to those repositories.

